# Isolated splenic tuberculosis in a patient with rheumatoid arthritis

**DOI:** 10.1016/j.idcr.2025.e02295

**Published:** 2025-06-19

**Authors:** Yuji Toyota, Akihiro Ito, Tadashi Ishida

**Affiliations:** Department of Respiratory Medicine, Ohara Healthcare Foundation, Kurashiki Central Hospital, 1-1-1 Miwa, Kurashiki, Okayama 710-8602, Japan

**Keywords:** Baricitinib, Methotrexate, Rheumatoid arthritis, Splenic tuberculosis, Tuberculosis

## Abstract

Isolated splenic tuberculosis (TB) is rare. Here, we report a case of isolated splenic TB in a 70-year-old man with rheumatoid arthritis (RA). The patient presented to the emergency department with a 3-day history of epigastric pain and hematemesis. For RA treatment, a combination of methotrexate (8 mg) and baricitinib (4 mg) had been initiated 2 years prior. Abdominal computed tomography (CT) scan revealed upper gastrointestinal hemorrhage and intrasplenic involvement. Following endoscopic hemostasis, we performed endoscopic ultrasound-guided fine-needle aspiration via the stomach due to suspected pancreatic cancer. At an outpatient follow-up visit 1 month later, fever and elevated C-reactive protein (9.02 mg/dL) levels were observed. CT imaging showed enlarged necrotic lymph nodes near the gastroesophageal junction, left mesentery of the colon, and the greater curvature of the pylorus, along with an increased low-density area in the spleen. Subsequently, upper gastrointestinal endoscopy and ultrasound-guided percutaneous fine-needle aspiration cytology were performed. Cultures from the abscesses tested positive for *Mycobacterium tuberculosis*, which was susceptible to isoniazid, rifampicin, ethambutol, and pyrazinamide. No lesions were identified, thus confirming a diagnosis of isolated splenic TB. Oral anti-TB treatment with four drugs (isoniazid, rifampicin, ethambutol, and pyrazinamide) was initiated. After 6 months of treatment, the splenic lesions had shrunk. Nine months after completing therapy, RA treatment was resumed without relapse. Therefore, early diagnosis and anti-TB treatment can successfully manage splenic TB without requiring splenectomy.

## Highlights

• Isolated splenic tuberculosis (TB) is rare, especially in the elderly with rheumatoid arthritis (RA).
• Fine-needle aspiration confirmed *Mycobacterium* tuberculosis infection.
• Six months of treatment shrank splenic lesions, no relapse after nine months.
• Early diagnosis and anti-TB treatment can successfully manage splenic TB without requiring splenectomy.

## Introduction

Japan is a medium-burden tuberculosis (TB) country, with 11,519 new TB cases reported in 2021, according to the Japan TB Surveillance Center's “Annual Report 2022” [1]. Extrapulmonary tuberculosis (TB) accounts for approximately 15 % of all cases [2]. Splenic TB is a rare disorder caused by the dissemination of pulmonary or biliary TB and was first reported by Coley in 1846 [3]. Splenic TB is frequently encountered in immunocompromised patients, especially those who are HIV-positive or in their disseminated form [4]. To the best of our knowledge, no reports of isolated splenic TB associated with rheumatoid arthritis (RA) exist. This report presents a rare case of isolated splenic TB accompanied by RA.

## Case report

**Fig. 1:**
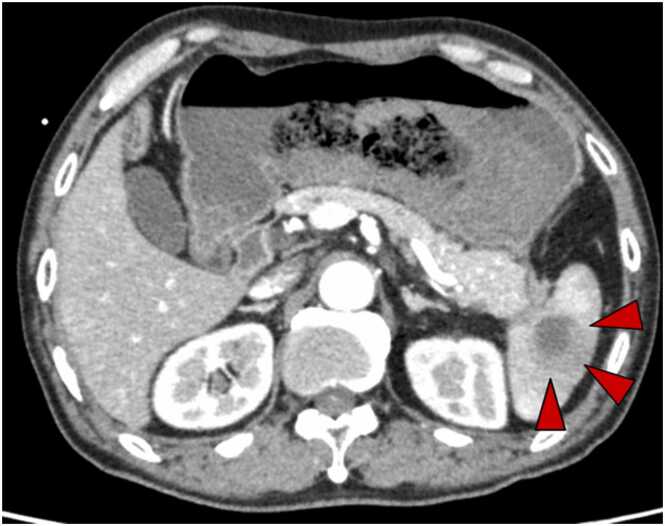
Contrast-enhanced abdominal CT at emergency department presentation revealed a 17 × 20 mm low density area within the spleen.

**Fig. 2:**
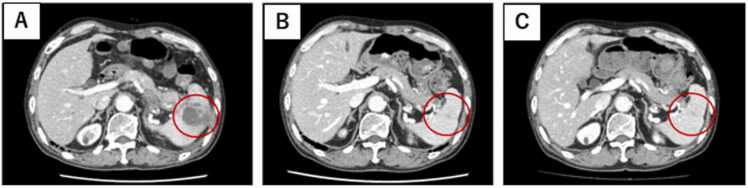
Abdominal computed tomography (CT) shows that anti-tuberculous treatment reduced the size of the lesion. A) CT on admission, B) CT at 5 months of treatment, C) CT at 9 months after the completion of treatment.

**Fig. 3:**
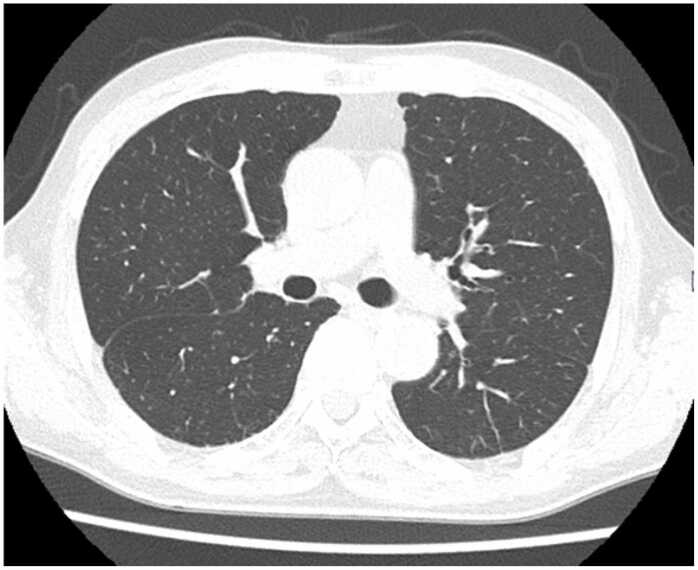
Initial chest X-ray and chest computed tomography (CT) at the time of diagnosis.

A 70-year-old man with RA presented to the emergency department with a 3-day history of epigastric pain and hematemesis. Five years prior, the patient was diagnosed with RA and started on methotrexate (MTX) therapy. Etanercept and tocilizumab were also used in combination with MTX, and a combination of MTX (8 mg) and baricitinib (4 mg) was initiated two years previously. We performed QuantiFERON® TB-Gold In-Tube testing (an interferon-gamma release assay; Cellestis, Ltd., Victoria, Australia) prior to initiating biologic agents. The result was negative. He denied any history of or exposure to tuberculosis. At the time of emergency medical examination, upper gastrointestinal bleeding was suspected based on low systolic blood pressure, low hemoglobin, and elevated blood urea nitrogen/creatinine levels. Contrast-enhanced computed tomography (CT) [Fig. 1] revealed a high-density area in the interior of the stomach, a low-density area in the spleen, and an irregularly shaped mass extending from the splenic hilum to the stomach. Pancreatic cancer metastasis and splenic infarction were considered differential diagnoses. After endoscopic hemostasis, we performed endoscopic ultrasound-guided fine-needle aspiration (EUS-FNA) via the stomach to evaluate the suspected pancreatic cancer [Fig. 2]. At an outpatient follow-up visit 1 month later, fever and an elevated C-reactive protein level (9.02 mg/dL) were observed. A CT scan [Fig. 2A] revealed enlarged necrotic lymph nodes in the gastroesophageal area, left mesentery of the colon, and the greater curvature of the pylorus, along with an increased low-density area in the spleen. Additionally, an abscess was found leaking into the stomach from the FNA site. The tumor marker levels were not elevated, and pancreatic cancer was ruled out. We repeated upper gastrointestinal endoscopy, which revealed an abscess leaking into the stomach; a splenic abscess was considered among the differential diagnoses. We started cefmetazole therapy following endoscopic abscess drainage. MTX and baricitinib were discontinued at the start of treatment. After administering cefmetazole, we observed no clinical improvement, so we escalated the treatment to meropenem. We performed ultrasound(US)-guided percutaneous FNAC to assess the lesion for tuberculosis, which was considered a likely differential diagnosis at that time. The patient continued to receive antibiotics for approximately 4 weeks, but no improvement in radiological or clinical findings was observed. Cultures from the abscesses tested positive for *Mycobacterium tuberculosis* which is susceptible to isoniazid (INH), rifampicin (RFP), ethambutol (EB), and pyrazinamide (PZA). Fecal, urine, sputum, and blood cultures were negative, and chest CT showed no evidence of pulmonary TB [Fig. 3], suggesting isolated splenic TB. The patient was started on oral anti-TB treatment with four drugs (INH, RFP, EB, and PZA) for 2 months and continued with two drugs (INH and RFP) for 7 months because of RA complications. This extended 9-month regimen, although longer than the standard WHO-recommended 6-month course for extrapulmonary TB, was chosen due to the patient's immunosuppressive status from active RA and prior use of baricitinib. According to the 2024 Japanese Tuberculosis Clinical Practice Guidelines, a 3-month treatment extension is weakly recommended in immunocompromised patients. This is based on evidence suggesting higher risk of relapse or complications in such populations [5]. The 9-month treatment period was completed without any adverse effects. The inflammatory response quickly decreased and clinical symptoms such as abdominal pain and fever subsided. We initially monitored the patient's RA without medication. However, one month after starting anti-TB therapy, the patient reported worsening joint pain. In response, we initiated prednisolone (15 mg). At 5-months, due to persistent symptoms, methotrexate (8 mg) was reintroduced. Although no significant increase in inflammatory markers such as CRP was observed (1.37 mg/dL at the time), the patient experienced gradually worsening joint pain. Based on clinical assessment and at the patient’s request, prednisolone was initiated. As symptoms persisted, methotrexate (8 mg) was reintroduced 5 months after starting anti-TB treatment. No formal disease activity scores such as DAS28 were obtained. The patient continued to progress well after MTX administration, and after 6 months of treatment, the splenic abscess had shrunk [Fig. 2B]. The patient is currently undergoing outpatient checkups to assess progress since the end of treatment. The patient had no recurrence and was doing well 9 months after treatment completion [Fig. 2C].

## Discussion

Herein, we report a case of isolated splenic TB in a patient with RA who received a combination of MTX and baricitinib. We detected splenic TB on imaging after the patient presented with upper gastrointestinal bleeding. Initially, pancreatic cancer metastasis and splenic infarction were considered differential diagnoses. However, because the imaging course was inconsistent with the above, including enlarged lymph nodes with necrosis, splenic TB was considered a differential diagnosis. The risk of active TB is higher in patients with RA, primarily due to the reactivation of latent TB. Several studies have reported a 2- to 10-fold increase in the risk of TB in patients with tumor necrosis factor (TNF) antagonist-naïve RA and a 2–30-fold increase in the risk of TB in patients exposed to TNF antagonists, compared with the general population [6]. JAK inhibitors have been reported to increase the risk of tuberculosis reactivation [7]. The TB relapse rate among patients treated with JAK inhibitors was 9.1 % [8]. Baricitinib, a selective JAK1 and JAK2 inhibitor, may predispose to tuberculosis by suppressing interferon-gamma (IFN-γ)-mediated signaling, which is essential for macrophage activation and granuloma maintenance [9]. This immunomodulatory effect compromises the host defense against *Mycobacterium tuberculosis*, potentially leading to reactivation of latent TB. Therefore, TB screening using interferon-gamma release assays (IGRAs), such as QuantiFERON-TB Gold, is recommended prior to initiating long-term JAK inhibitor therapy, and serial testing may be considered in high-risk populations.

The risk of tuberculosis varies among immunosuppressive agents used in RA. Table 1 summarizes the TB risk associated with JAK inhibitors, TNF inhibitors, and other biologics. TNF inhibitors are considered high-risk [10], while JAK inhibitors carry an intermediate risk [10], [11]. Among JAK inhibitors, tofacitinib at high doses (10 mg twice daily) has shown increased TB incidence [10], and baricitinib is associated with a moderate risk [12]. IL-6 inhibitors and B cell-depleting agents such as rituximab are generally associated with lower risks [10]. In this case, over 2 years had passed since the last etanercept treatment, and the patient was receiving MTX and baricitinib. Patients infected with human immunodeficiency virus (HIV) or immunocompromised patients are at a high risk of developing splenic TB [3], the association between splenic TB and RA is unclear. An Indian study on the clinical profile of patients with splenic involvement in TB revealed that 50 % of the patients with splenic TB were infected with HIV; however, no RA complications were reported [13]. To the best of our knowledge, only one case report of isolated splenic TB associated with RA has been published [14]. Although isolated splenic TB is rare in patients with RA, immunosuppression by MTX may contribute to its development. Isolated splenic TB has a few specific symptoms, making early diagnosis difficult, and splenectomy can be performed for diagnostic purposes [15]. In isolated splenic TB, the macronodular form is often observed on CT or US [16]. In the early stages, radiological findings on contrast-enhanced imaging, including CT, are similar to those of an abscess, whereas calcification is a typical finding in the advanced stages. The differential diagnosis of the macronodular form includes metastases, abscesses, and primary malignancy [16]. In the present case, no characteristic findings such as calcification were present; therefore, splenic TB was not considered a differential diagnosis at the initial examination. However, the diagnosis was reconsidered based on the absence of elevated tumor markers and the presence of necrotic lymph nodes in the surrounding area. Therefore, splenic TB was included in the differential diagnosis. Consequently, ultrasound-guided FNAC was performed to avoid splenectomy. In addition to splenectomy, FNAC is a useful diagnostic method. In 31 cases using FNAC for patients with suspected abdominal TB, 58 % had a confirmed diagnosis, with eight cases using FNAC for the spleen, reporting a diagnostic accuracy of 87.5 % [17]. In the present case, pancreatic cancer metastasis and splenic infarction were the first differential diagnoses. However, based on the clinical course of the patient, splenic TB was identified as a differential diagnosis, which was confirmed by FNAC. Previous reports described several cases of splenectomy that may be avoided by considering TB when treating splenic lesions in patients with rheumatoid arthritis.

**Table 1:** Comparative risk of tuberculosis (TB) associated with immunosuppressive agents used in rheumatoid arthritis (RA).

Drug Class	Representative Agents	Estimated TB Risk	Notes
TNF inhibitors	Infliximab, Adalimumab	High (Peto OR: 3.98, 95 % CI: 2.30–6.88)	Strong association with TB reactivation. Mandatory IGRA screening recommended before initiation.
JAK inhibitors	Tofacitinib (10 mg), Baricitinib	Moderate to High (Dose-dependent)	Tofacitinib 10 mg BID shows significantly higher TB risk (Peto OR: 7.39, 95 % CI: 2.00–27.31).
IL−6 inhibitors	Tocilizumab	Low to Moderate (Peto OR: 5.98, 95 % CI: 0.80–44.89)	Lower risk compared to TNF inhibitors; not statistically significant. TB screening still recommended.
B cell depleting agents	Rituximab	Not assessed	Not included in the cited meta-analysis; caution advised in endemic regions.
CTLA−4 Ig	Abatacept	Not assessed	Not evaluated in this meta-analysis; limited reports suggest lower TB risk.

## Conclusions

The diagnosis of splenic TB remains challenging because its clinical and radiological features have a wide spectrum, mimicking several other diseases. Splenic TB often appears as part of a miliary TB lesion but may be observed as solitary splenic TB in immunosuppressed patients. Early diagnosis and treatment of splenic TB may prevent the need for splenectomy. When a patient with rheumatoid arthritis presents with splenic involvement, TB should be considered in the differential diagnosis, even in the absence of multi-organ involvement.

## CRediT authorship contribution statement

**Tadashi Ishida:** Writing – review & editing. **Akihiro Ito:** Writing – review & editing. **Yuji Toyota:** Writing – original draft.

## Ethical approval

Ethical approval for this study was obtained from the Ethics Committee of Kurashiki Central Hospital (Approval No. 4699).

## Author contribution

Yuji Toyota was responsible for drafting the work, conception and design of the work, and acquisition, analysis, and interpretation of the data for the work. Akihiro Ito critically revised the manuscript for important intellectual content. Tadashi Ishida approved the final manuscript for publication.

## Informed consent and patient details

The written informed consent was obtained from the patient for publication.

## Funding sources

None.

## Author Statement

I, Yuji Toyota, the corresponding author, certify that all authors have made substantial contributions to the conception and design of the work, as well as to the acquisition, analysis, and interpretation of data. All authors were involved in drafting the manuscript, revising it critically for important intellectual content, and gave final approval of the version to be published. Each author agrees to be accountable for all aspects of the work in ensuring that questions related to the accuracy or integrity of any part of the work are appropriately investigated and resolved.

## Declaration of Generative AI and AI-assisted technologies in the writing process

The authors affirm that generative AI and AI-assisted technologies were not used in the writing process.

## Declaration of Competing Interest

The authors declare that they have no known competing financial interests or personal relationships that could have appeared to influence the work reported in this paper.
